# Exploiting Cooperative Downlink NOMA in D2D Communications

**DOI:** 10.3390/s23083958

**Published:** 2023-04-13

**Authors:** Ashish Rauniyar, Olav N. Østerbø, Jan Erik Håkegård, Paal Engelstad

**Affiliations:** 1 Sustainable Communication Technologies, SINTEF Digital, 7034 Trondheim, Norway; 2 Telenor Research, 1360 Oslo, Norway; 3 Autonomous Systems and Sensor Technologies Research Group, Department of Technology Systems, University of Oslo, 0316 Oslo, Norway

**Keywords:** internet of things, bidirectional, device-to-device (D2D), non-orthogonal multiple access (NOMA), relaying, outage probability, ergodic capacity

## Abstract

We propose and investigate a bidirectional device-to-device (D2D) transmission scheme that exploits cooperative downlink non-orthogonal multiple access (NOMA) (termed as BCD-NOMA). In BCD-NOMA, two source nodes communicate with their corresponding destination nodes via a relaying node while exchanging bidirectional D2D messages simultaneously. BCD-NOMA is designed for improved outage probability (OP) performance, high ergodic capacity (EC) and high energy efficiency by allowing two sources to share the same relaying node for data transmission to their corresponding destination nodes while also facilitating bidirectional D2D communications exploiting downlink NOMA. Simulation and analytical expressions of the OP, EC and ergodic sum capacity (ESC) under both perfect and imperfect successive interference cancellation (SIC) are used to demonstrate the effectiveness of BCD-NOMA compared to conventional schemes.

## 1. Introduction

The use of wireless communication and data transfer is expanding rapidly worldwide. With the advent of new technologies such as holographic communications, Metaverse, and Internet of Things (IoT) applications in smart cities has triggered unprecedented exponential growth in data traffic. It has been predicted that global wireless traffic will surpass an incredible 5016 exabytes by 2030 [1]. Addressing these needs necessitates innovative solutions to meet the ever-increasing demand for wireless data. To satisfy these applications’ capacity and connectivity requirements, non-orthogonal multiple access (NOMA) is regarded as a potential key candidate for the upcoming sixth generation (6G) networks due to its high spectral efficiency [2,3,4]. NOMA can support a large number of users and provide a high data rate transmission by allowing multiple users to share the same frequency band and time slot. Additionally, NOMA can enhance the reliability of communication and reduce the latency in network communication [5]. In contrast to orthogonal multiple access (OMA) approaches such as orthogonal frequency-division multiple access (OFDMA) and time-division multiple access (TDMA), NOMA does not rely on orthogonal resources to separate the users [6,7]. Instead, it transmits different signals to different users simultaneously via superposition coding [8,9]. In particular, different users can share the same orthogonal resources in NOMA by assigning them to different power levels and using successive interference cancellation (SIC) at the receiver side [10,11,12]. NOMA can be used to support heterogeneous services by allowing users with different channel conditions to share the same resources, which can support a variety of services with different quality-of-service (QoS) requirements [13]. Furthermore, cooperative NOMA (CNOMA) with a decode-and-forward (DF) relaying scheme has been introduced in the literature to further enhance the performance of NOMA systems [14,15]. Cooperative NOMA leverages the proximity of near users to a base station (BS) to enhance the communication quality of cell edge users that are located further away. In this setup, the users closest to the BS act as relays for those situated further away. By doing so, cooperative NOMA can improve the overall reliability and capacity of the network [16]. The combination of cooperative relaying and NOMA is particularly effective because of the SIC technique. SIC allows for the near users to decode and remove the signals intended for the far users, as the information for the far users is already known to them. By leveraging this knowledge, cooperative NOMA can reduce interference and increase the data rate for all users.

On the other hand, device-to-device (D2D) communication can further improve the spectral efficiency (SE) and has already been used in NOMA settings [17,18,19]. Instead of utilizing a central BS, D2D communication enables devices to connect with one another directly. In areas where the BS’s signal is poor, D2D communication can improve coverage [20,21]. Moreover, D2D communication allows for devices to communicate with each other directly, which can reduce latency and power consumption compared to traditional cellular communication [22,23].

In [24], a D2D-aided transmission strategy utilizing uplink NOMA is proposed where two similar gain near users and a far user are served in a cooperative scenario where nearby users are able to communicate directly with the BS. In contrast, cell edge users far from the BS need the help of one of the nearby users to facilitate communication. A cooperative NOMA relaying strategy (NOMA-RS), where two sources communicate with their corresponding destinations in parallel over the same frequency band via a common relay, is proposed in [25]. In order to maximize the uplink energy efficiency and throughput, a joint sub-channel and power allocation algorithm based on Kuhn-Munkres (KM) technique for D2D communication in NOMA is considered in [26]. A strategy for the power allocation optimization by using the sub-gradient method in NOMA-enabled D2D communication is proposed in [27]. The authors formulated the optimization problem to maximize the sum data rate while adhering to the QoS requirements for cellular users and transmit power restrictions for D2D communication. To address the issue of maximizing network throughput in D2D Communications With NOMA, the authors in [28] have utilized a swarm intelligence approach called the Whale Optimization Algorithm (WOA). This approach investigates a joint resource allocation optimization problem involving user clustering, power control, and D2D mode selection. Similarly, to increase the total achievable rate, and cellular coverage, a power allocation strategy for NOMA-based D2D systems is introduced in [29]. Moreover, incorporating bidirectional communication in NOMA can further boost spectral efficiency [30]. A bidirectional D2D communication in cooperative uplink NOMA is explored in [31], where two far users can exchange D2D messages while sending their messages to the BS via a cooperative relaying node. A hybrid cellular and bidirectional D2D cooperative NOMA system, where two users require downlink signals from the BS for energy harvesting and data transmission while simultaneously exchanging information with each other, is investigated in [32]. While these research works have explored D2D-enabled NOMA communication, the effective integration of cooperative downlink NOMA and bidirectional D2D communication that can further boost the system’s capacity and energy efficiency remains relatively unexplored. Cooperative downlink NOMA and bidirectional D2D communications can enable a new range of applications and services, such as peer-to-peer content sharing, multiplayer gaming, etc.

On the basis of the existing research on NOMA-enabled D2D communications, there is a significant interest in developing schemes that can further boost the system’s capacity and energy efficiency. Motivated by the works in [24,25,31], we propose and investigate a bidirectional D2D transmission scheme that exploits cooperative downlink NOMA scheme (termed as BCD-NOMA). Unlike previous works, BCD-NOMA is designed for improved outage probability (OP) performance, high ergodic capacity (EC), and high energy efficiency by allowing two source nodes to share the same relaying node for data transmission to their corresponding destination nodes while also facilitating bidirectional D2D communications between them by exploiting downlink NOMA. The main contributions of this work are outlined as follows:

1. We investigate and propose a BCD-NOMA transmission strategy that uses cooperative downlink NOMA, allowing users to transmit data to the shared relay and D2D device in tandem and transfer their decoded signal at the relay in parallel as well to their respective destination nodes.
2. We derive the analytical expressions for the OP, EC, and ergodic sum capacity (ESC) under both perfect SIC (pSIC) and imperfect SIC (ipSIC) scenarios and verify them with the simulation results.
3. We verify the effectiveness of the BCD-NOMA scheme in terms of OP, ESC and average energy efficiency through simulations and mathematical analysis over schemes such as orthogonal multiple access (OMA), cooperative NOMA with OMA (CNOMA-OMA) and other conventional schemes.

The paper is structured as follows: Section 2 presents the proposed BCD-NOMA system and its corresponding channel model. In Section 3, the OP performance of the BCD-NOMA system is examined, including its analytical expressions. Section 4 focuses on the evaluation of the ergodic capacity and ergodic sum capacity of the proposed BCD-NOMA system, along with their corresponding analytical derivations. To support the performance evaluations, numerical results and discussions are presented in Section 5. These results demonstrate the effectiveness of the proposed BCD-NOMA system in enhancing ESC, average energy efficiency and improved OP performance. Finally, the conclusions drawn from the study are presented in Section 6, summarizing the main contributions of the research and highlighting potential areas for future work.

## 2. System Model

**Figure 1:**
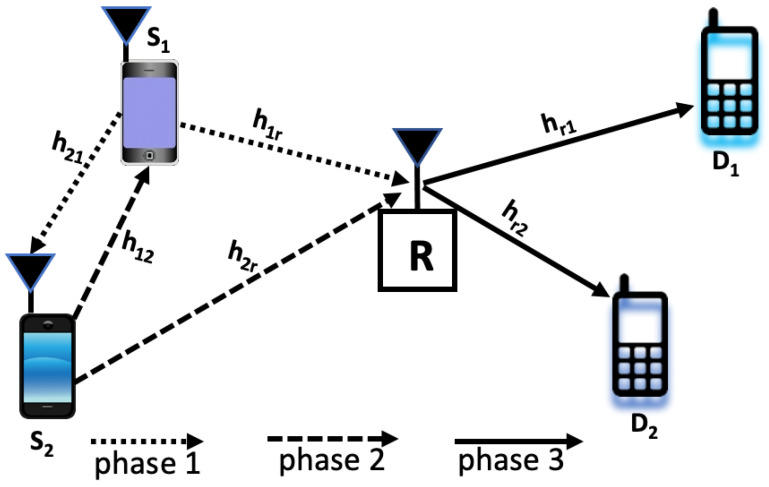
System model.

We consider a cooperative downlink NOMA scenario as shown in Figure 1, where two source-destination pairs, $S_{1}-D_{1}$ and $S_{2}-D_{2}$, communicate through a shared relay node *R*. *R* is considered a single antenna half-duplex relay employing a DF strategy. Due to deep fading or blocking, there is no direct connection between the $S_{1}-D_{1}$ and $S_{2}-D_{2}$ links [18]. Thus, their data exchange relies on the relay node *R*. Further, to facilitate D2D bidirectional communications, the source nodes $S_{1}$ and $S_{2}$ communicate with each other by exchanging D2D messages. We have also assumed that channel state information (CSI) is perfectly known to the receivers and is in line with previous works such as [33,34]. The nodes are assumed to be equipped with a single antenna and operate in the half-duplex mode. The channel between any two nodes is subjected to the independent Rayleigh block fading plus additive white Gaussian noise. Furthermore, $h_{1r}$∼$CN(0,\lambda_{1r}$) is the complex channel coefficient between $S_{1}$ and *R* node with zero mean and variance $\lambda_{1r}$. Similarly, $h_{2r}$∼$CN(0,\lambda_{2r})$, $h_{r1}$∼$CN(0,\lambda_{r1})$, and $h_{r2}$∼$CN(0,\lambda_{r2})$ are the complex channel coefficient for the links $S_{2}-R$, $R-D_{1}$, and $R-D_{2}$, respectively. In addition, $h_{12}$∼$CN(0,\lambda_{h_{12}})$ is the complex channel coefficient between $S_{1}$ and $S_{2}$ nodes with zero mean and variance $\lambda_{h_{12}}$. The channel between $S_{1}$ and $S_{2}$ node is considered reciprocal. Therefore, $h_{12}\approx h_{21}$. Furthermore, without the loss of generality, we consider that $\lambda_{12}>\lambda_{1r}>\lambda_{2r}$ and $\lambda_{r2}>\lambda_{r1}$. So, it is expected that $|h_{12}{|}^{2}>|h_{1r}{|}^{2}>{\left|h_{2r}\right|}^{2}$, and $|h_{r2}{|}^{2}>{\left|h_{r1}\right|}^{2}$. Though $\lambda_{12}>\lambda_{1r}>\lambda_{2r}$ and $\lambda_{r2}>\lambda_{r1}$ may not guarantee $|h_{12}{|}^{2}>|h_{1r}{|}^{2}>{\left|h_{2r}\right|}^{2}$, and $|h_{r2}{|}^{2}>{\left|h_{r1}\right|}^{2}$, it is a simple but effective strategy to employ this assumption under statistical channel state information [35]. This is also in line with previous works such as [24,36]. As shown in Figure 1, the data transmission of the BCD-NOMA scheme is divided into three communication phases which are described below.

### 2.1. Phase-1 ($t_{1}$)

In this phase, $S_{1}$ transmits a composite NOMA signal $x_{t_{1}}=\sqrt{a_{1}P_{S_{1}}}x_{S_{1}}+\sqrt{a_{2}P_{S_{1}}}x_{S_{1}-S_{2}}$, where $x_{S_{1}}$ is the downlink message, $x_{S_{1}-S_{2}}$ is the D2D message, $P_{S_{1}}$ is the transmit power of $S_{1}$ node, and $a_{1}$, $a_{2}$ are the NOMA power allocation coefficients with $a_{1}>a_{2}$ and $a_{1}+a_{2}=1$. Following downlink NOMA, the received signal-to-interference-plus-noise ratio (SINR) at $S_{2}$ node is given as:

(1) $$\gamma_{S_{2}\to S_{1}-R}^{x_{S_{1}}}=\frac{\rho a_{1}{\left|h_{12}\right|}^{2}}{\rho a_{2}{\left|h_{12}\right|}^{2}+1},$$

(2) $$\gamma_{S_{2}\to S_{1}-S_{2}}^{S_{1}-S_{2}}=\frac{\rho a_{2}{\left|h_{12}\right|}^{2}}{\rho a_{1}{\left|\hat{h_{12}}\right|}^{2}+1},$$

where $\rho =\frac{P_{S_{1}}}{\sigma^{2}}$, $\sigma^{2}$ denotes noise variance, and $\hat{h_{12}}∼CN\left(0,\xi \lambda_{12}\right)$, and the parameter ξ(0≤ξ≤1) denotes the level of residual interference because of SIC imperfection. In particular, ξ=1 and ξ=0 represent imperfect and perfect SIC cases, respectively.

The received SINR at *R* is given as:

(3) $$\gamma_{R\to S_{1}}^{x_{S_{1}}}=\frac{\rho a_{1}{\left|h_{1r}\right|}^{2}}{\rho a_{2}{\left|h_{1r}\right|}^{2}+1},$$

### 2.2. Phase-2 ($t_{2}$)

In this phase, $S_{2}$ transmits a composite NOMA signal $x_{t_{2}}=\sqrt{a_{3}P_{S_{2}}}x_{S_{2}}+\sqrt{a_{4}P_{S_{2}}}x_{S_{2}-S_{1}}$, where $x_{S_{2}}$ is the downlink message, $x_{S_{2}-S_{1}}$ is the D2D message, $P_{S_{2}}$ is the transmit power of $S_{2}$ node, respectively, and $a_{3}$, $a_{4}$ are the NOMA power allocation coefficients with $a_{3}>a_{4}$ and $a_{3}+a_{4}=1$. Following downlink NOMA, the received SINR at $S_{1}$ node can be given as:

(4) $$\gamma_{S_{1}\to S_{2}-R}^{x_{S_{2}}}=\frac{\rho a_{3}{\left|h_{12}\right|}^{2}}{\rho a_{4}{\left|h_{12}\right|}^{2}+1},$$

(5) $$\gamma_{S_{1}\to S_{2}-S_{1}}^{S_{2}-S_{1}}=\frac{\rho a_{4}{\left|h_{12}\right|}^{2}}{\rho a_{3}{\left|\hat{h_{12}}\right|}^{2}+1},$$

where $\rho =\frac{P_{S_{2}}}{\sigma^{2}}$.

The received SINR at *R* in this phase is given as:

(6) $$\gamma_{R\to S_{2}}^{x_{S_{2}}}=\frac{\rho a_{3}{\left|h_{2r}\right|}^{2}}{\rho a_{4}{\left|h_{2r}\right|}^{2}+1},$$

### 2.3. Phase-3 ($t_{3}$)

In this phase, *R* transmits a composite NOMA signal $x_{t_{3}}=\sqrt{b_{1}P_{r}}\hat{x_{S_{1}}}+\sqrt{b_{2}P_{r}}\hat{x_{S_{2}}}$, where $\hat{x_{S_{1}}}$ is the decoded downlink message of $S_{1}$ for the destination $D_{1}$, $\hat{x_{S_{2}}}$ is the decoded downlink message of $S_{2}$ for the destination $D_{2}$, $P_{r}$ is the transmit power of the relay node *R*, and $b_{1}$, $b_{2}$ are the NOMA power allocation coefficients with $b_{1}>b_{2}$ and $b_{1}+b_{2}=1$. Again, following downlink NOMA, the received SINR at $D_{2}$ node can be given as:

(7) $$\gamma_{D_{2}\to S_{1}}^{x_{S_{1}}}=\frac{\rho_{r}b_{1}{\left|h_{r2}\right|}^{2}}{\rho_{r}b_{2}{\left|h_{r2}\right|}^{2}+1},$$

(8) $$\gamma_{D_{2}\to S_{2}}^{x_{S_{2}}}=\frac{\rho_{r}b_{2}{\left|h_{r2}\right|}^{2}}{\rho_{r}b_{1}{\left|\hat{h_{r2}}\right|}^{2}+1},$$

where $\rho_{r}=\frac{P_{r}}{\sigma^{2}}$, and $\hat{h_{r2}}∼CN\left(0,\xi \lambda_{r2}\right)$.

The received SINR at the $D_{1}$ node is given as:

(9) $$\gamma_{D_{1}\to S_{1}}^{x_{S_{1}}}=\frac{\rho_{r}b_{1}{\left|h_{r1}\right|}^{2}}{\rho_{r}b_{2}{\left|h_{r1}\right|}^{2}+1},$$

## 3. Outage Probability Analysis

The outage probability is defined as the probability that the end-to-end signal-to-noise ratio (SNR) at the destination falls below a given SNR threshold. This section presents the outage probability analysis for our BCD-NOMA system.

### 3.1. Outage Probability of $S_{1}$ Node Associated with the $x_{S_{1}}$ Symbol

The $S_{1}$ node associated with the $x_{S_{1}}$ symbol will be in an outage if any of the following conditions hold true:

1. $S_{2}$ cannot decode the symbol $x_{S_{1}}$ in phase-1.
2. *R* cannot decode the symbol $x_{S_{1}}$ in phase-1.
3. $D_{2}$ cannot decode the downlink message of $S_{1}$ transmitted from *R* in phase-3.
4. $D_{1}$ cannot decode the downlink message of $S_{1}$ transmitted from *R* in phase-3.

The above conditions for the outage probability of $S_{1}$ node associated with the $x_{S_{1}}$ symbol can be expressed as:

(10) $$\begin{array}{cc} P_{S_{1}}^{out} & =1-(\mathrm{Pr}(\gamma_{S_{2}\to S_{1}-R}^{x_{S_{1}}}\geq \gamma_{S_{1}}^{T})\cap \mathrm{Pr}(\gamma_{R\to S_{1}}^{x_{S_{1}}}\geq \gamma_{S_{1}}^{T})\cap \mathrm{Pr}(\gamma_{D_{2}\to S_{1}}^{x_{S_{1}}}\geq \gamma_{S_{1}}^{T})\cap \\ \; & \;\mathrm{Pr}(\gamma_{D_{1}\to S_{1}}^{x_{S_{1}}}\geq \gamma_{S_{1}}^{T})) \end{array}$$

where $\gamma_{i}^{T},=2^{3R_{i}}-1$ is the lower SINR threshold value, i.e., the outage probability, $i\in \{S_{1},S_{2},S_{1}-S_{2},S_{2}-S_{1}\}$ with $R_{i}$ denoting the target data rate of the users.

Let, $|h_{12}{|}^{2}=Z_{1},|h_{1r}{|}^{2}=X_{1},{\left|h_{r2}\right|}^{2}=Y_{2}$ and $|h_{r1}{|}^{2}=Y_{1}$.

Substituting these in Equation (10), we obtain

$$\begin{array}{cc} \; & P_{S_{1}}^{out}=1-(\mathrm{Pr}\left(Z_{1}\geq \frac{\gamma_{S_{1}}^{T}}{\rho (a_{1}-\gamma_{S_{1}}^{T}a_{2})}\right)\mathrm{Pr}\left(X_{1}\geq \frac{\gamma_{S_{1}}^{T}}{\rho (a_{1}-\gamma_{S_{1}}^{T}a_{2})}\right)\mathrm{Pr}\left(Y_{2}\geq \frac{\gamma_{S_{1}}^{T}}{\rho_{r}\left(b_{1}-\gamma_{S_{1}}^{T}b_{2}\right)}\right)\\ \; & \mathrm{Pr}\left(Y_{1}\geq \frac{\gamma_{S_{1}}^{T}}{\rho_{r}\left(b_{1}-\gamma_{S_{1}}^{T}b_{2}\right)}\right))\\ \; & P_{S_{1}}^{out}=1-e^{-\frac{\lambda_{12}\gamma_{S_{1}}^{T}}{\rho (a_{1}-\gamma_{S_{1}}^{T}a_{2})}}e^{-\frac{\lambda_{1r}\gamma_{S_{1}}^{T}}{\rho (a_{1}-\gamma_{S_{1}}^{T}a_{2})}}e^{-\frac{\lambda_{r2}\gamma_{S_{1}}^{T}}{\rho_{r}\left(b_{1}-\gamma_{S_{1}}^{T}b_{2}\right)}}e^{-\frac{\lambda_{r1}\gamma_{S_{1}}^{T}}{\rho_{r}\left(b_{1}-\gamma_{S_{1}}^{T}b_{2}\right)}} \end{array}$$

After rearranging the terms, the closed-form analytical expression for the outage probability of $S_{1}$ node associated with the $x_{S_{1}}$ symbol can be expressed as:

(11) $$P_{S_{1}}^{out-Ana}=1-e^{-\frac{\left(\lambda_{12}+\lambda_{1r}\right)\gamma_{S_{1}}^{T}}{\rho (a_{1}-\gamma_{S_{1}}^{T}a_{2})}}e^{-\frac{\left(\lambda_{r2}+\lambda_{r1}\right)\gamma_{S_{1}}^{T}}{\rho (b_{1}-\gamma_{S_{1}}^{T}b_{2})}}$$

As it can be seen in Equation (11), it does not contain any ξ term. Therefore, the outage probability of $S_{1}$ node associated with symbol $x_{1}$ is not affected by ipSIC.

### 3.2. Outage Probability of $S_{2}$ Node Associated with the $x_{S_{2}}$ Symbol

The $S_{2}$ node associated with the $x_{S_{2}}$ symbol will be in an outage if any of the following conditions hold true:

1. $S_{1}$ cannot decode the symbol $x_{S_{2}}$ in phase-2.
2. *R* cannot decode the symbol $x_{S_{2}}$ in phase-2.
3. $D_{2}$ cannot decode the downlink message of $S_{2}$ transmitted from *R* in phase-3.

The above conditions for the outage probability of $S_{2}$ node associated with the $x_{S_{2}}$ symbol can be expressed as:

(12) $$P_{S_{2}}^{out}=1-\left(\mathrm{Pr}(\gamma_{S_{1}\to S_{2}-R}^{x_{S_{2}}}\geq \gamma_{S_{2}}^{T})\cap \mathrm{Pr}(\gamma_{R\to S_{2}}^{x_{S_{2}}}\geq \gamma_{S_{2}}^{T})\cap \mathrm{Pr}(\gamma_{D_{2}\to S_{2}}^{x_{S_{2}}}\geq \gamma_{S_{2}}^{T})\right)$$

Let, $|h_{12}{|}^{2}=Z_{1},{\left|h_{2r}\right|}^{2}=X_{2}$, and $|h_{r2}{|}^{2}=Y_{2}$.

Substituting these in Equation (12), we obtain

$P_{S_{2}}^{out}=1-\mathrm{Pr}(Z_{1}\geq \frac{\gamma_{S_{2}}^{T}}{\rho (a_{3}-\gamma_{S_{2}}^{T}a_{4})})\mathrm{Pr}(X_{2}\geq \frac{\gamma_{S_{2}}^{T}}{\rho (a_{3}-\gamma_{S_{2}}^{T}a_{4})})\mathrm{Pr}(Y_{2}\geq \frac{\gamma_{S_{2}}^{T}}{\rho_{r}\left(b_{2}-\gamma_{S_{2}}^{T}\xi b_{1}\right)})$

$P_{S_{2}}^{out}=1-e^{-\frac{\lambda_{12}\gamma_{S_{2}}^{T}}{\rho (a_{3}-\gamma_{S_{2}}^{T}a_{4})}}e^{-\frac{\lambda_{2r}\gamma_{S_{2}}^{T}}{\rho (a_{3}-\gamma_{S_{2}}^{T}a_{4})}}e^{-\frac{\lambda_{r2}\gamma_{S_{2}}^{T}}{\rho_{r}\left(b_{2}-\gamma_{S_{2}}^{T}\xi b_{1}\right)}}$

After rearranging the terms, the closed-form analytical expression for the outage probability of $S_{2}$ node associated with the $x_{S_{2}}$ symbol can be expressed as:

(13) $$P_{S_{2}}^{out-Ana}=1-e^{-\frac{\left(\lambda_{12}+\lambda_{2r}\right)\gamma_{S_{2}}^{T}}{\rho (a_{3}-\gamma_{S_{2}}^{T}a_{4})}}e^{-\frac{\lambda_{r2}\gamma_{S_{2}}^{T}}{\rho_{r}\left(b_{2}-\gamma_{S_{2}}^{T}\xi b_{1}\right)}}$$

### 3.3. Outage Probability of $S_{1}$ D2D Message Associated with the $x_{S_{1}-S_{2}}$ Symbol

The $S_{1}$ D2D message associated with the $x_{S_{1}-S_{2}}$ symbol will be in an outage if the $S_{2}$ node cannot decode it during phase-1. Therefore, the outage probability of $S_{1}$ D2D message associated with the $x_{S_{1}-S_{2}}$ symbol can be expressed as:

(14) $$P_{D2D,S1-S2}^{out}=1-\mathrm{Pr}\left(\gamma_{S_{2}\to S_{1}-S_{2}}^{S_{1}-S_{2}}\geq \gamma_{S_{1}-S_{2}}^{T}\right)$$

Let, $|h_{12}{|}^{2}=Z_{1}$. Substituting this in Equation (14), we obtain

$P_{D2D,S1-S2}^{out}=1-\mathrm{Pr}\left(\frac{\rho a_{2}Z_{1}}{\rho a_{1}\xi Z_{1}+1}\geq \gamma_{S_{1}-S_{2}}^{T}\right)$

$P_{D2D,S1-S2}^{out}=1-\mathrm{Pr}(Z_{1}\geq \frac{\gamma_{S_{1}-S_{2}}^{T}}{\rho (a_{2}-\gamma_{S_{1}-S_{2}}^{T}\xi a_{1})})$

Therefore, the closed-form analytical expression for the outage probability of of $S_{1}$ D2D message associated with the $x_{S_{1}-S_{2}}$ symbol can be expressed as:

(15) $$P_{D2D,S1-S2}^{out-Ana}=1-e^{-\frac{\lambda_{12}\gamma_{S_{1}-S_{2}}^{T}}{\rho (a_{2}-\gamma_{S_{1}-S_{2}}^{T}\xi a_{1})}}$$

### 3.4. Outage Probability of $S_{2}$ D2D Message Associated with the $x_{S_{2}-S_{1}}$ Symbol

The $S_{2}$ D2D message associated with the $x_{S_{2}-S_{1}}$ symbol will be in an outage if the $S_{1}$ node cannot decode it during phase-2. Therefore, the outage probability of $S_{2}$ D2D message associated with the $x_{S_{2}-S_{1}}$ symbol can be expressed as:

(16) $$P_{D2D,S2-S1}^{out}=1-\mathrm{Pr}(\gamma_{S_{1}\to S_{2}-S_{1}}^{S_{2}-S_{1}}\geq \gamma_{S_{2}-S_{1}}^{T})$$

Let, $|h_{12}{|}^{2}=Z_{1}$. Substituting this in Equation (16), we obtain

$P_{D2D,S2-S1}^{out}=1-\mathrm{Pr}\left(\frac{\rho a_{4}Z_{1}}{\rho a_{3}\xi Z_{1}+1}\geq \gamma_{S_{2}-S_{1}}^{T}\right)$

$P_{D2D,S2-S1}^{out}=1-\mathrm{Pr}\left(Z_{1}\geq \frac{\gamma_{S_{2}-S_{1}}^{T}}{\rho (a_{4}-\gamma_{S_{2}-S_{1}}^{T}\xi a_{3})}\right)$

Therefore, the closed-form analytical expression for the outage probability of $S_{2}$ D2D message associated with the $x_{S_{2}-S_{1}}$ symbol can be expressed as:

(17) $$P_{D2D,S2-S1}^{out-Ana}=1-e^{-\frac{\lambda_{12}\gamma_{S_{2}-S_{1}}^{T}}{\rho (a_{4}-\gamma_{S_{2}-S_{1}}^{T}\xi a_{3})}}$$

## 4. Ergodic Capacity Analysis

In this section, we will analyze the achievable data rate of each of the nodes and the achievable sum rate of the proposed BCD-NOMA system.

### 4.1. Achievable Rate of $S_{1}$ Node Associated with the $x_{S_{1}}$ Symbol

According to our system model, the achievable capacity for the $S_{1}$ node associated with the $x_{S_{1}}$ symbol is given by:

(18) $$C_{x_{1}}=E\left[\frac{1}{3}\log_{2}\left(1+\min\left(\gamma_{S_{2}\to S_{1}-R}^{x_{S_{1}}},\gamma_{R\to S_{1}}^{x_{S_{1}}},\gamma_{D_{2}\to S_{1}}^{x_{S_{1}}},\gamma_{D_{1}\to S_{1}}^{x_{S_{1}}}\right)\right)\right]$$

where *E* [·] denotes the statistical expectation operator, and the factor $\frac{1}{3}$ represents that three transmission phases are involved in the BCD-NOMA system.

**Theorem 1.**  *The closed-form analytical expression for the achievable capacity for the $S_{1}$ node associated with the $x_{S_{1}}$ symbol can be expressed as:*

(19) $$\begin{array}{c} C_{x_{1}}^{Ana}=\frac{1}{3\ln2}\left(e^{\frac{K+D}{a_{1}+a_{2}}}E_{1}\left(\frac{K+D}{a_{1}+a_{2}}\right)-e^{\frac{K+D}{a_{2}}}E_{1}\left(\frac{K+D}{a_{2}}\right)\right) \end{array}$$

where $K=\frac{\left(\lambda_{12}+\lambda_{1r}\right)}{\rho },$ and $D=\frac{\left(\lambda_{r1}+\lambda_{r2}\right)}{\rho_{r}}$ and $E_{1}\left(\cdot \right)$ is exponential integral of order 1.

**Proof.**  Let $\gamma =\min\left(\gamma_{S_{2}\to S_{1}-R}^{x_{S_{1}}},\gamma_{R\to S_{1}}^{x_{S_{1}}},\gamma_{D_{2}\to S_{1}}^{x_{S_{1}}},\gamma_{D_{1}\to S_{1}}^{x_{S_{1}}}\right)$.

The cumulative distributive function (CDF) of γ can be given as:

$F_{\gamma }\left(\gamma \right)=1-e^{\frac{-(\lambda_{12}+\lambda_{1r})\gamma }{\rho (a_{1}-\gamma a_{2})}}e^{\frac{-(\lambda_{r2}+\lambda_{r1})\gamma }{\rho_{r}\left(b_{1}-\gamma b_{2}\right)}}$

By using $\int_{x=0}^{\infty }\log_{2}\left(1+x\right)f_{X}\left(x\right)dx=\frac{1}{\ln2}\int_{x=0}^{\infty }\frac{1-F_{X}\left(x\right)}{1+x}dx,$ the analytical expression for EC of $S_{1}$ node, i.e., $C_{x_{1}}^{Ana}$ can be computed as:

$C_{x_{1}}^{Ana}=\frac{1}{3\ln2}\int_{\gamma =0}^{\infty }\frac{1}{1+\gamma }e^{\frac{-(\lambda_{12}+\lambda_{1r})\gamma }{\rho (a_{1}-\gamma a_{2})}}e^{\frac{-(\lambda_{r2}+\lambda_{r1})\gamma }{\rho_{r}\left(b_{1}-\gamma b_{2}\right)}}d\gamma$

Now, for mathematical tractability, we assume that $a_{1}=b_{1}$ and $a_{2}=b_{2}$. Other than this, for different cases, the proof remains the same as of Theorem 2 and can be derived by following the similar steps as in the proof of Theorem 2.

Therefore, the above expression becomes:

$$C_{x_{1}}^{Ana}=\frac{1}{3\ln2}\int_{\gamma =0}^{\frac{a_{1}}{a_{2}}}\frac{e^{-\left(K+D\right)\frac{\gamma }{\left(a_{1}-\gamma a_{2}\right)}}}{1+\gamma }d\gamma$$

where $K=\frac{\left(\lambda_{12}+\lambda_{1r}\right)}{\rho },$ and $D=\frac{\left(\lambda_{r1}+\lambda_{r2}\right)}{\rho_{r}}$.

Now, by changing variable $x=\frac{\gamma }{a_{1}-\gamma a_{2}}$ and applying partial fraction decomposition, we obtain

$C_{x_{1}}^{Ana}=\frac{1}{3\ln2}\left(\int_{x=0}^{\infty }\frac{\left(a_{1}+a_{2}\right)}{(1+\left(a_{1}+a_{2}\right)x}e^{-(K+D)x}dx-\int_{x=0}^{\infty }\frac{a_{2}}{\left(1+a_{2}x\right)}e^{-(K+D)x}dx\right)$

The above integrals can be easily expressed in terms of the exponential integral function $E_{1}\left(x\right)$ by:

$C_{x_{1}}^{Ana}=\frac{1}{3\ln2}\left(e^{\frac{K+D}{a_{1}+a_{2}}}E_{1}\left(\frac{K+D}{a_{1}+a_{2}}\right)-e^{\frac{K+D}{a_{2}}}E_{1}\left(\frac{K+D}{a_{2}}\right)\right)$

This completes the proof of Theorem 1. □

As it can be seen in Equation (19), it does not contain any ξ term. Therefore, the EC of $S_{1}$ node associated with symbol $x_{1}$ is not affected by ipSIC.

### 4.2. Achievable Rate of $S_{2}$ Node Associated with the $x_{S_{2}}$ Symbol

Similarly, the achievable capacity for the $S_{2}$ node associated with the $x_{S_{2}}$ symbol is given by:

(20) $$C_{x_{2}}=E\left[\frac{1}{3}\log_{2}\left(1+\min\left(\gamma_{S_{1}\to S_{2}-R}^{x_{S_{2}}},\gamma_{R\to S_{2}}^{x_{S_{2}}},\gamma_{D_{2}\to S_{2}}^{x_{S_{2}}}\right)\right)\right]$$

**Theorem 2.**  *The closed-form analytical expression for the achievable capacity for the $S_{2}$ node associated with the $x_{S_{2}}$ symbol can be expressed as:*

(21) $$\begin{array}{c} C_{x_{2}}^{Ana}=\left\{\begin{array}{c} \mathrm{when}\;\frac{a_{3}}{a_{4}}<\frac{b_{2}}{\xi b_{1}},\\ \frac{e^{\frac{Lb_{2}-Ma_{3}}{a_{4}b_{2}-a_{3}\xi b_{1}}}}{3\ln2}\left(J\left(\frac{b_{2}\left(a_{3}+a_{4}\right)}{a_{3}\left(b_{2}+\xi b_{1}\right)},\frac{Ma_{3}}{a_{4}b_{2}-a_{3}\xi b_{1}},\frac{Lb_{2}}{a_{4}b_{2}-a_{3}\xi b_{1}}\right)-J\left(\frac{b_{2}a_{4}}{\xi b_{1}a_{3}},\frac{Ma_{3}}{a_{4}b_{2}-a_{3}\xi b_{1}},\frac{Lb_{2}}{a_{4}b_{2}-a_{3}\xi b_{1}}\right)\right),\\ \mathrm{when}\;\frac{a_{3}}{a_{4}}>\frac{b_{2}}{\xi b_{1}},\\ \frac{e^{\frac{Lb_{2}-Ma_{3}}{a_{4}b_{2}-a_{3}\xi b_{1}}}}{3\ln2}\left(J\left(\frac{a_{3}\left(\xi b_{1}+b_{2}\right)}{b_{2}\left(a_{3}+a_{4}\right)},\frac{Lb_{2}}{\xi b_{1}a_{3}-b_{2}a_{4}},\frac{Ma_{3}}{\xi b_{1}a_{3}-b_{2}a_{4}}\right)-J\left(\frac{a_{3}\xi b_{1}}{a_{4}b_{2}},\frac{Lb_{2}}{\xi b_{1}a_{3}-b_{2}a_{4}},\frac{Ma_{3}}{\xi b_{1}a_{3}-b_{2}a_{4}}\right)\right),\\ \mathrm{when}\;\frac{a_{3}}{a_{4}}=\frac{b_{2}}{\xi b_{1}},\\ \frac{1}{3\ln2}\left(e^{\frac{Lb_{2}+Ma_{3}}{\left(a_{3}+a_{4}\right)b_{2}}}E_{1}\left(\frac{Lb_{2}+Ma_{3}}{\left(a_{3}+a_{4}\right)b_{2}}\right)-e^{\frac{Lb_{2}+Ma_{3}}{a_{4}b_{2}}}E_{1}\left(\frac{Lb_{2}+Ma_{3}}{a_{4}b_{2}}\right)\right) \end{array}\right. \end{array}$$

where $L=\frac{\left(\lambda_{12}+\lambda_{2r}\right)}{\rho }$, $M=\frac{\lambda_{r2}}{\rho_{r}}$ and $J\left(a,b,c\right)=e^{-(\frac{c}{a}-ab)}E_{1}(\frac{(a-1)c}{a})-\sum_{i=1}^{\infty }\frac{1}{a^{i-1}}E_{i}\left(c\right)(e^{ab}-\sum_{k=0}^{i-1}\frac{{\left(ab\right)}^{k}}{k!})$.

**Proof.**  Let $Z=\min(\gamma_{S_{1}\to S_{2}-R}^{x_{S_{2}}},\gamma_{R\to S_{2}}^{x_{S_{2}}},\gamma_{D_{2}\to S_{2}}^{x_{S_{2}}})$.

The CDF of *Z* can be given as:

$F_{Z}\left(z\right)=1-e^{\frac{-(\lambda_{12}+\lambda_{2r})z}{\rho (a_{3}-za_{4})}}e^{\frac{-\lambda_{r2}z}{\rho_{r}\left(b_{2}-z\xi b_{1}\right)}}$

By using $\int_{x=0}^{\infty }\log_{2}\left(1+x\right)f_{X}\left(x\right)dx=\frac{1}{\ln2}\int_{x=0}^{\infty }\frac{1-F_{X}\left(x\right)}{1+x}dx,$

The analytical expression for EC of $S_{2}$ node associated with symbol $x_{2}$, i.e., $C_{x_{2}}^{Ana}$ can be computed as:

(22) $$C_{x_{2}}^{Ana}=\frac{1}{3\ln2}\int_{z=0}^{\infty }\frac{1}{1+z}e^{\frac{-(\lambda_{12}+\lambda_{2r})z}{\rho (a_{3}-za_{4})}}e^{\frac{-\lambda_{r2}z}{\rho_{r}\left(b_{2}-z\xi b_{1}\right)}}dz$$

In the above equation, z should be less than $\min(\frac{a_{3}}{a_{4}},\frac{b_{2}}{\xi b_{1}})$.

Therefore, we now evaluate the above expression in three different cases, as explained below.

**Case 1:** When $\frac{a_{3}}{a_{4}}<\frac{b_{2}}{\xi b_{1}}$, the above $C_{x_{2}}^{Ana}$ expression in Equation (22) becomes:

$C_{x_{2}}^{Ana}=\frac{1}{3\ln2}\int_{z=0}^{\frac{a_{3}}{a_{4}}}\frac{1}{1+z}e^{\frac{-(\lambda_{12}+\lambda_{2r})z}{\rho (a_{3}-za_{4})}}e^{\frac{-\lambda_{r2}z}{\rho_{r}\left(b_{2}-z\xi b_{1}\right)}}dz$

Now, by changing variable $x=\frac{z}{a_{3}-za_{4}}$ and applying partial fraction decomposition, we obtain

$$C_{x_{2}}^{Ana}=\frac{1}{3\ln2}\left(\int_{x=0}^{\infty }\frac{\left(a_{3}+a_{4}\right)}{\left(1+\left(a_{3}+a_{4}\right)x\right)}e^{-Lx-M\frac{a_{3}x}{b_{2}+\left(a_{4}b_{2}-a_{3}\xi b_{1}\right)x}}dx-\int_{x=0}^{\infty }\frac{a_{4}}{\left(1+a_{4}x\right)}e^{-Lx-M\frac{a_{3}x}{b_{2}+\left(a_{4}b_{2}-a_{3}\xi b_{1}\right)x}}dx\right)$$

where $L=\frac{\left(\lambda_{12}+\lambda_{2r}\right)}{\rho }$ and $M=\frac{\lambda_{r2}}{\rho_{r}}$.

Now, we make substitution $u=1+\frac{(a_{4}b_{2}-a_{3}\xi b_{1})x}{b_{2}}$ and transforming the integrals to:

$C_{x_{2}}^{Ana}=\frac{e^{\frac{Lb_{2}-Ma_{3}}{a_{4}b_{2}-a_{3}\xi b_{1}}}}{3\ln2}\left(\int_{u=1}^{\infty }\frac{-\frac{b_{2}\left(a_{3}+a_{4}\right)}{a_{3}\left(b_{2}+\xi b_{1}\right)}}{1-\frac{b_{2}\left(a_{3}+a_{4}\right)}{a_{3}\left(b_{2}+\xi b_{1}\right)}u}e^{\frac{Ma_{3}}{(a_{4}b_{2}-a_{3}\xi b_{1})u}-\frac{Lb_{2}u}{a_{4}b_{2}-a_{3}\xi b_{1}}}du-\int_{u=1}^{\infty }\frac{-\frac{b_{2}a_{4}}{\xi b_{1}a_{3}}}{1-\frac{b_{2}a_{4}}{\xi b_{1}a_{3}}u}e^{\frac{Ma_{3}}{(a_{4}b_{2}-a_{3}\xi b_{1})u}-\frac{Lb_{2}u}{a_{4}b_{2}-a_{3}\xi b_{1}}}du\right)$

We can express the above integral in the form $J\left(a,b,c\right)=-\int_{y=1}^{\infty }\frac{ae^{\frac{b}{y}-cy}}{1-ay}dy$.

Hence,

(23) $$\begin{array}{c} C_{x_{2}}^{Ana}=\frac{e^{\frac{Lb_{2}-Ma_{3}}{a_{4}b_{2}-a_{3}\xi b_{1}}}}{3\ln2}(J\left(\frac{b_{2}\left(a_{3}+a_{4}\right)}{a_{3}\left(b_{2}+\xi b_{1}\right)},\frac{Ma_{3}}{a_{4}b_{2}-a_{3}\xi b_{1}},\frac{Lb_{2}}{a_{4}b_{2}-a_{3}\xi b_{1}}\right)\\ -J\left(\frac{b_{2}a_{4}}{\xi b_{1}a_{3}},\frac{Ma_{3}}{a_{4}b_{2}-a_{3}\xi b_{1}},\frac{Lb_{2}}{a_{4}b_{2}-a_{3}\xi b_{1}}\right)) \end{array}$$

Now, after some algebraic manipulation J(a,b,c) can be easily expressed as: $J\left(a,b,c\right)=e^{-(\frac{c}{a}-ab)}E_{1}(\frac{(a-1)c}{a})-\sum_{i=1}^{\infty }\frac{1}{a^{i-1}}E_{i}\left(c\right)(e^{ab}-\sum_{k=0}^{i-1}\frac{{\left(ab\right)}^{k}}{k!})$.

Substituting the expression for J(a,b,c) in Equation (22), gives the analytical expression for $C_{x_{2}}^{Ana}$ for Case 1.

**Case 2:** When $\frac{a_{3}}{a_{4}}>\frac{b_{2}}{\xi b_{1}}$, the expression in Equation (22) becomes:

$C_{x_{2}}^{Ana}=\frac{1}{3\ln2}\int_{z=0}^{\frac{b_{2}}{\xi b_{1}}}\frac{1}{1+z}e^{\frac{-(\lambda_{12}+\lambda_{2r})z}{\rho (a_{3}-za_{4})}}e^{\frac{-\lambda_{r2}z}{\rho_{r}\left(b_{2}-z\xi b_{1}\right)}}dz$

Following the similar steps as in Case 1, the analytical expression for $C_{x_{2}}^{Ana}$ for Case 2 can be given as:

$C_{x_{2}}^{Ana}=\frac{e^{\frac{Lb_{2}-Ma_{3}}{a_{4}b_{2}-a_{3}\xi b_{1}}}}{3\ln2}\left(J\left(\frac{a_{3}\left(\xi b_{1}+b_{2}\right)}{b_{2}\left(a_{3}+a_{4}\right)},\frac{Lb_{2}}{\xi b_{1}a_{3}-b_{2}a_{4}},\frac{Ma_{3}}{\xi b_{1}a_{3}-b_{2}a_{4}}\right)-J\left(\frac{a_{3}\xi b_{1}}{a_{4}b_{2}},\frac{Lb_{2}}{\xi b_{1}a_{3}-b_{2}a_{4}},\frac{Ma_{3}}{\xi b_{1}a_{3}-b_{2}a_{4}}\right)\right)$

**Case 3:** When $\frac{a_{3}}{a_{4}}=\frac{b_{2}}{\xi b_{1}}$, the expression in Equation (22) can be easily expressed in terms of the exponential integral as:

$C_{x_{2}}^{Ana}=\frac{1}{3\ln2}\left(e^{\frac{Lb_{2}+Ma_{3}}{\left(a_{3}+a_{4}\right)b_{2}}}E_{1}\left(\frac{Lb_{2}+Ma_{3}}{\left(a_{3}+a_{4}\right)b_{2}}\right)-e^{\frac{Lb_{2}+Ma_{3}}{a_{4}b_{2}}}E_{1}\left(\frac{Lb_{2}+Ma_{3}}{a_{4}b_{2}}\right)\right)$

This completes the proof of Theorem 2. □

Since the analytical expression for $C_{x_{2}}^{Ana}$, as shown in Cases 1 and 2, contains an infinite summation term in J(a,b,c). We now provide the convergence analysis of the infinite summation term in J(a,b,c).

Since, $J\left(a,b,c\right)=e^{-(\frac{c}{a}-ab)}E_{1}(\frac{(a-1)c}{a})-\sum_{i=1}^{\infty }\frac{1}{a^{i-1}}E_{i}\left(c\right)(e^{ab}-\sum_{k=0}^{i-1}\frac{{\left(ab\right)}^{k}}{k!})$, we first define the truncated exponential sum $S_{i}\left(x\right)=\sum_{k=i}^{\infty }\frac{x^{k}}{k!}$.

Now, J(a,b,c) can be further expressed as:

$J\left(a,b,c\right)=e^{-(\frac{c}{a}-ab)}E_{1}(\frac{(a-1)c}{a})-\underset{A_{1}}{\underbrace{\sum_{i=1}^{\infty }\frac{E_{i}\left(c\right)}{a^{i-1}}S_{i}\left(ab\right)}}$.

We have to show that the above infinite summation term $A_{1}$ in J(a,b,c) converges.

Now, $A_{1}=\sum_{i=1}^{\infty }\frac{E_{i}\left(c\right)}{a^{i-1}}S_{i}\left(ab\right)=e^{ab}E_{1}\left(c\right)+\sum_{i=2}^{\infty }\frac{E_{i}\left(c\right)}{a^{i-1}}S_{i}\left(ab\right)$.

Since we have $S_{i}\left(ab\right)\leq e^{ab}$ and $E_{i}\left(c\right)\leq E_{i}\left(c=0\right)=\frac{1}{i-1}$.

This implies that $\frac{E_{i}\left(c\right)}{a^{i-1}}S_{i}\left(ab\right)\leq \frac{e^{ab}}{\left(i-1\right)a^{i-1}}\leq \frac{e^{ab}}{a^{i-1}}$ for i≥2, and it follows that the series $A_{1}$ is convergent since the series $\sum_{i=2}^{\infty }\frac{e^{ab}}{a^{i-1}}$ is convergent for a>1.

We now also derive the analytical expression for $C_{x_{2}}^{Ana}$ at a high SNR region.

At high SNR, it holds that $Z\approx \min(\frac{a_{3}}{a_{4}},\gamma_{D_{2}\to S_{2}}^{x_{S_{2}}})$.

Therefore following [37], $C_{x_{2}}^{Ana,\rho \to \infty }$ at high SNR can be approximated as:

(24) $$C_{x_{2}}^{Ana,\rho \to \infty }\approx \int_{\gamma =0}^{\frac{a_{3}}{a_{4}}}\frac{e^{-\lambda_{r2}\frac{\gamma }{\rho_{r}\left(b_{2}-\gamma \xi b_{1}\right)}}}{1+\gamma }d\gamma$$

Now, by changing variable $x=\frac{\gamma }{b_{2}-\gamma \xi b_{1}}$, applying partial fraction decomposition, and transforming the integrals in exponential form, we finally obtain:

(25) $$C_{x_{2}}^{Ana,\rho \to \infty }\approx \frac{1}{3\ln2}(e^{P}(E_{1}\left(P\right)-E_{1}\left(Q\right))-e^{R}(E_{1}\left(R\right)-E_{1}\left(S\right)))$$

where $P=\frac{\lambda_{r2}}{\rho_{r}\left(b_{2}+\xi b_{1}\right)}$, $Q=\frac{\lambda_{r2}}{\rho_{r}}\left(\frac{1}{\left(b_{2}+\xi b_{1}\right)}+\frac{a_{3}}{\left(a_{4}b_{2}-a_{3}\xi b_{1}\right)}\right)$, $R=\frac{\lambda_{r2}}{\rho_{r}\xi b_{1}}$, and $S=\frac{\lambda_{r2}}{\rho_{r}}\left(\frac{1}{\xi b_{1}}+\frac{a_{3}}{\left(a_{4}b_{2}-a_{3}\xi b_{1}\right)}\right)$.

### 4.3. Achievable Rate of $S_{1}$ D2D Message Associated with the $x_{S_{1}-S_{2}}$ Symbol

The achievable capacity for the $S_{1}$ D2D message associated with symbol $x_{S_{1}-S_{2}}$ is given as:

(26) $$C_{D2D}^{x_{S_{1}-S_{2}}}=E\left[\frac{1}{3}\log_{2}\left(1+\gamma_{S_{2}\to S_{1}-S_{2}}^{S_{1}-S_{2}}\right)\right]$$

**Theorem 3.**  *The closed-form analytical expression for the achievable capacity $S_{1}$ D2D message associated with the $x_{S_{1}-S_{2}}$ symbol can be expressed as:*

(27) $$\begin{array}{c} C_{D2D}^{x_{S_{1}-S_{2}}-Ana}=\frac{1}{3\ln2}\left(e^{\frac{\lambda_{12}}{\rho (a_{2}+\xi a_{1})}}E_{1}\left(\frac{\lambda_{12}}{\rho (a_{2}+\xi a_{1})}\right)-e^{\frac{\lambda_{12}}{\rho \xi a_{1}}}E_{1}\left(\frac{\lambda_{12}}{\rho \xi a_{1}}\right)\right) \end{array}$$

**Proof.**  Let $\gamma =\gamma_{S_{2}\to S_{1}-S_{2}}^{S_{1}-S_{2}}$.

The CDF of $\gamma =\gamma_{S_{2}\to S_{1}-S_{2}}^{S_{1}-S_{2}}$ can be expressed as:

$F_{\gamma }\left(\gamma \right)=1-e^{\frac{-\lambda_{12}\gamma }{\rho (a_{2}-\gamma \xi a_{1})}}$.

By using $\int_{x=0}^{\infty }\log_{2}\left(1+x\right)f_{X}\left(x\right)dx=\frac{1}{\ln2}\int_{x=0}^{\infty }\frac{1-F_{X}\left(x\right)}{1+x}dx,$ the analytical expression for EC of $S_{1}$ D2D message associated with symbol $x_{S_{1}-S_{2}}$, i.e., $C_{D2D}^{x_{S_{1}-S_{2}}-Ana}$ can be computed as:

$C_{D2D}^{x_{S_{1}-S_{2}}-Ana}=\frac{1}{3\ln2}\int_{\gamma =0}^{\infty }\frac{1}{1+\gamma }e^{\frac{-\lambda_{12}\gamma }{\rho (a_{2}-\gamma \xi a_{1})}}d\gamma$

Let, $x=\frac{\gamma }{\left(a_{2}-\gamma \xi a_{1}\right)}\to \gamma =\frac{a_{2}x}{1+\xi a_{1}x}\to d\gamma =\frac{a_{2}}{{\left(1+\xi a_{1}x\right)}^{2}}dx$

$C_{D2D}^{x_{S_{1}-S_{2}}-Ana}=\frac{1}{3\ln2}\int_{x=0}^{\infty }\frac{e^{\frac{-\lambda_{12}x}{\rho }}}{1+(a_{2}+\xi a_{1})x}\frac{a_{2}}{\left(1+\xi a_{1}x\right)}dx$

Now, applying partial fraction decomposition, we obtain

$C_{D2D}^{x_{S_{1}-S_{2}}-Ana}=\frac{\left(\xi a_{1}+a_{2}\right)}{3\ln2}\underset{I_{1}}{\underbrace{\int_{x=0}^{\infty }\frac{e^{\frac{-\lambda_{12}x}{\rho }}}{1+(a_{2}+\xi a_{1})x}dx}}-\frac{\left(\xi a_{1}\right)}{3\ln2}\underset{I_{2}}{\underbrace{\int_{x=0}^{\infty }\frac{e^{\frac{-\lambda_{12}x}{\rho }}}{\left(1+\xi a_{1}x\right)}dx}}$

After some straightforward algebraic manipulation, $I_{1}$ can be expressed as:

$I_{1}=\frac{e^{\frac{\lambda_{12}}{\rho (a_{2}+\xi a_{1})}}}{\left(a_{2}+\xi a_{1}\right)}E_{1}\left(\frac{\lambda_{12}}{\rho (a_{2}+\xi a_{1})}\right)$.

Similarly, $I_{2}$ can be expressed as: $I_{2}=\frac{e^{\frac{\lambda_{12}}{\rho \xi a_{1}}}}{\xi a_{1}}E_{1}\left(\frac{\lambda_{12}}{\rho \xi a_{1}}\right)$.

Substituting the value for $I_{1}$ and $I_{2}$, gives the final analytical expression for $C_{D2D}^{x_{S_{1}-S_{2}}-Ana}$ as:

$C_{D2D}^{x_{S_{1}-S_{2}}-Ana}=\frac{1}{3\ln2}\left(e^{\frac{\lambda_{12}}{\rho (a_{2}+\xi a_{1})}}E_{1}\left(\frac{\lambda_{12}}{\rho (a_{2}+\xi a_{1})}\right)-e^{\frac{\lambda_{12}}{\rho \xi a_{1}}}E_{1}\left(\frac{\lambda_{12}}{\rho \xi a_{1}}\right)\right)$

This completes the proof of Theorem 3. □

### 4.4. Achievable Rate of $S_{2}$ D2D Message Associated with the $x_{S_{2}-S_{1}}$ Symbol

Finally, the achievable capacity for the $S_{2}$ D2D message associated with symbol $x_{S_{2}-S_{1}}$ is given as:

(28) $$C_{D2D}^{x_{S_{2}-S_{1}}}=E\left[\frac{1}{3}\log_{2}\left(1+\gamma_{S_{1}\to S_{2}-S_{1}}^{S_{2}-S_{1}}\right)\right]$$

**Theorem 4.**  *The closed-form analytical expression for the achievable capacity $S_{2}$ D2D message associated with the $x_{S_{2}-S_{1}}$ symbol can be expressed as:*

(29) $$\begin{array}{c} C_{D2D}^{x_{S_{2}-S_{1}}-Ana}=\frac{1}{3\ln2}\left(e^{\frac{\lambda_{12}}{\rho (a_{4}+\xi a_{3})}}E_{1}\left(\frac{\lambda_{12}}{\rho (a_{4}+\xi a_{3})}\right)-e^{\frac{\lambda_{12}}{\rho \xi a_{3}}}E_{1}\left(\frac{\lambda_{12}}{\rho \xi a_{3}}\right)\right) \end{array}$$

**Proof.**  Following the similar steps as for the analytical derivation of $C_{D2D}^{x_{S_{1}-S_{2}}-Ana}$ in Theorem 3, the final analytical expression for $C_{D2D}^{x_{S_{2}-S_{1}}-Ana}$ can be derived as in Equation (29). □

### 4.5. ESC of the BCD-NOMA System

The ESC of the BCD-NOMA system is given by:

(30) $$\begin{array}{cc} C_{Sys} & =C_{x_{1}}+C_{x_{2}}+C_{D2D}^{x_{S_{1}-S_{2}}}+C_{D2D}^{x_{S_{2}-S_{1}}}\\ \; & =(18)+(20)+(26)+(28) \end{array}$$

Combining Equations (19), (21), (27) and (29) gives the analytical expression for the ESC of the BCD-NOMA system.

Furthermore, we may find an approximation for high SNR by expanding both $e^{x}$ and $E_{1}\left(x\right)$ to the first order, i.e., by taking $e^{x}\approx 1+x$ and $E_{1}\left(x\right)\approx -\gamma -\ln\left(x\right)+x$. For $C_{x_{2}}$ approximation, we also used the high SNR approximation as shown in Equation (25).

## 5. Results and Discussions

For simplicity, we consider the normalized distances of the nodes and the relay node as in [31], i.e., $d_{S_{1}-R}=0.25$, $d_{S_{2}-R}=0.50$, $d_{R-D_{1}}=0.50$, $d_{R-D_{2}}=0.25$ and $d_{S_{1}-S_{2}}=0.20$. Furthermore, path loss exponent vs. is set to 4, $\rho =\rho_{r}$ and fixed NOMA power allocation method is used [25], i.e., $a_{1}=0.7$, $a_{2}=0.3$, $a_{3}=0.8$, $a_{4}=0.2$, $b_{1}=0.7$, and $b_{2}=0.3$. We average over $10^{6}$ randomly generated Rayleigh block fading channels in MATLAB to run the Monte-Carlo simulation and obtain the simulation results. The list of simulation parameters are given in Table 1.

OMA, hybrid CNOMA-OMA and NOMA relaying scheme (NOMA-RS) of Ref. [25] are used as benchmarks to show the performance gain of the proposed BCD-NOMA scheme. Six time slots are required to complete the data transmission in the OMA scheme. In the hybrid CNOMA-OMA scheme, four time slots are required, i.e., $S_{1}$ and $S_{2}$ utilize uplink NOMA in the first time slot to send their data to the relay node, and the relay node transmits the data to the destination nodes utilizing downlink NOMA in the second time slot. Two time slots are required by $S_{1}$ and $S_{2}$ for their D2D message transmission, which is accomplished by using OMA. NOMA-RS scheme of Ref. [25] utilizes two time slots without D2D data transmission.

**Figure 2:**
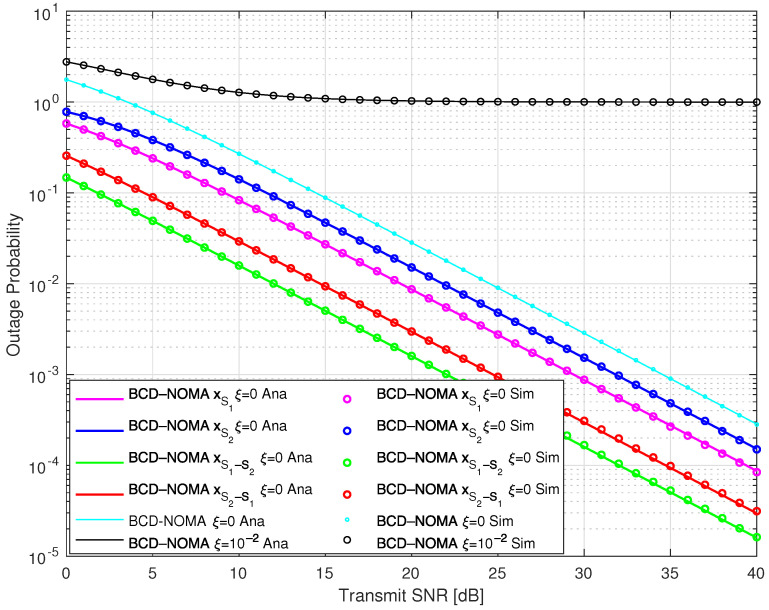
Outage probability of BCD-NOMA scheme.

**Figure 3:**
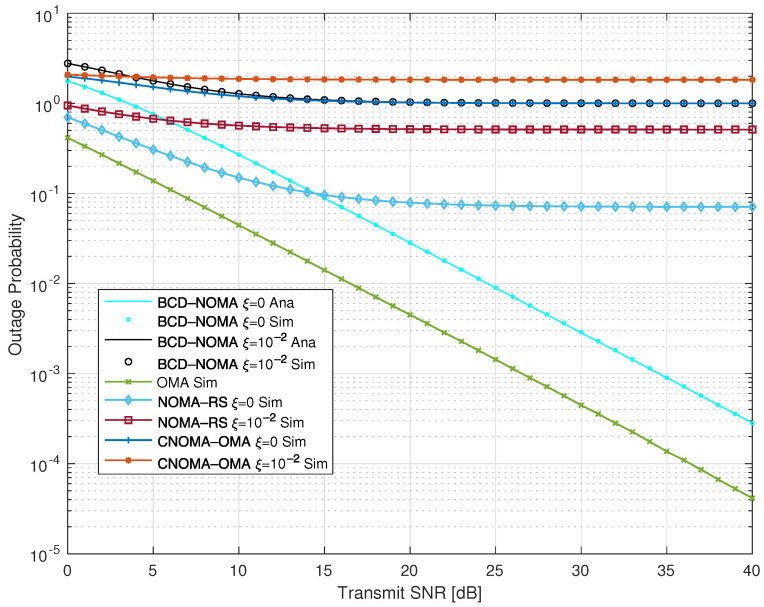
Outage probability comparison of BCD-NOMA scheme with benchmarks.

**Figure 4:**
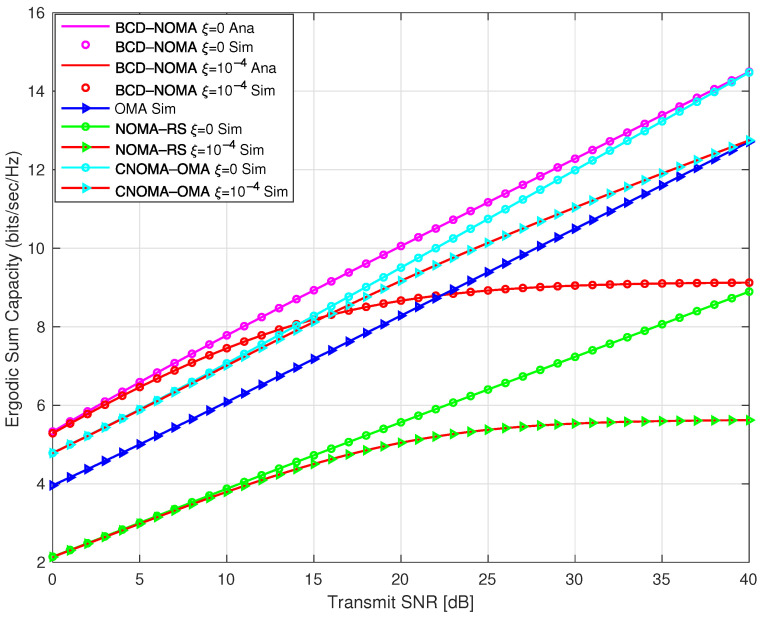
ESC comparison of BCD-NOMA scheme and benchmarks.

**Figure 5:**
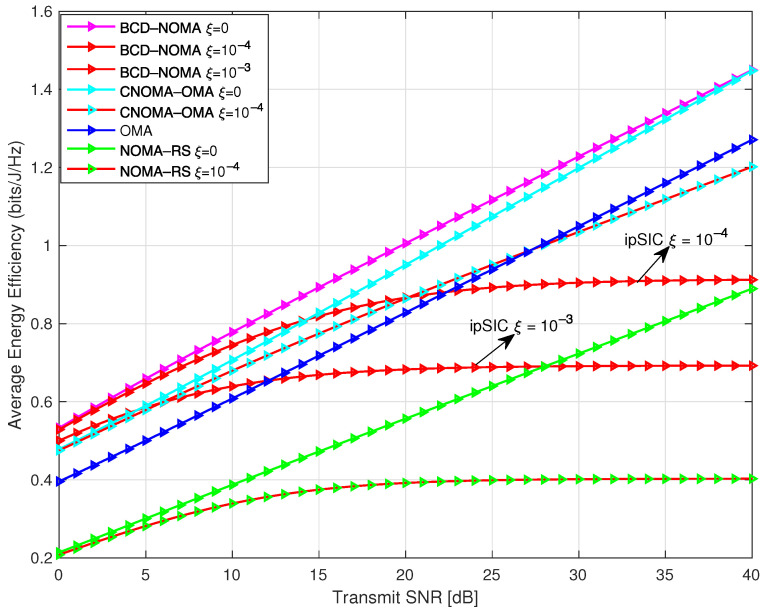
Energy efficiency comparison of BCD-NOMA scheme and benchmarks, where $P_{S_{1}}=P_{S_{2}}=P_{r}=10$ W.

In Figure 2 and Figure 3, we examine the OP performance of our proposed BCD-NOMA system and compare it with the benchmarks. Specifically, in Figure 2, we plot the OP of each of the symbols, i.e., $x_{S_{1}}$, $x_{S_{2}}$, $x_{S_{1}-S_{2}}$, $x_{S_{2}-S_{1}}$ and the OP of the BCD-NOMA system under pSIC and ipSIC. We observe that the $x_{S_{2}}$ symbol has the worst outage performance, and $x_{S_{1}-S_{2}}$ has the best outage performance compared to other symbols in the BCD-NOMA system. Moreover, ipSIC also tends to increase the outage probability in the BCD-NOMA system, as indicated in Figure 2. To further gain insight into the OP performance, we compare the OP performance of the BCD-NOMA with different benchmarks, as shown in Figure 3. We observe that the OP performance of the BCD-NOMA system under pSIC is better than the CNOMA-OMA scheme and shows a comparable OP performance to the CNOMA-OMA scheme under ipSIC, especially at a transmitting SNR higher than 10 dB. In addition, the OP performance of BCD-NOMA under pSIC is better than the NOMA-RS scheme of Ref. [25], especially at a transmitting SNR greater than 15 dB. The NOMA-RS scheme shows a better OP than the BCD-NOMA at a transmitting SNR less than 15 dB. The reason is that in the NOMA-RS scheme, no D2D communication is considered. Therefore, a better outage performance is expected for the NOMA-RS scheme even under the ipSIC case, as clearly depicted in Figure 2. Moreover, the OMA scheme shows improved outage performance compared to our BCD-NOMA scheme, which is due to the fact that in the OMA scheme, the nodes transmit at full power, which further improves its OP performance compared to our BCD-NOMA scheme. However, this can be compensated with the increased ESC and energy efficiency performance as indicated in Figure 4 and Figure 5, respectively. Additionally, there is a close match between the analytical and simulation results, demonstrating the integrity of the analytical expressions we deduced.

In Figure 4, we compare the ESC of the BCD-NOMA scheme with the benchmark schemes. We observe that the ESC performance of the BCD-NOMA scheme, especially for the pSIC case, outperforms all the benchmark schemes against all transmit SNR ρ values. The ESC performance gain between pSIC and ipSIC depends on the level of residual interference. It can be clearly observed at medium to high ρ values. Moreover, at higher ρ values, the hybrid CNOMA-OMA scheme has almost comparable ESC performance with the BCD-NOMA scheme. This is because in a hybrid CNOMA-OMA scheme, the D2D nodes, i.e., $S_{1}$ and $S_{2}$, transmit at full transmit power in the last two time slots, thereby increasing their overall ESC performance at high ρ values. In addition, a close match between the analytical and simulation results is observed in Figure 4, which indicates that our derived analytical expressions for the BCD-NOMA scheme are intact.

Energy efficiency (EE) is an important metric to check the performance of next-generation wireless systems. The EE metric is defined as the ratio of the total data rate of the system over the total energy consumed by the system [38]. In Figure 5, we compare the average EE of the BCD-NOMA scheme against all the benchmark schemes. We notice that our proposed BCD-NOMA scheme is more energy-efficient compared to all other schemes. Moreover, we can also observe that ipSIC tends to lower the EE performance and thus significantly impacts the ESC performance. Therefore, more intelligent SIC techniques can further enhance the ESC and EE performance of the proposed BCD-NOMA system.

## 6. Conclusions and Future Works

We proposed and investigated a BCD-NOMA scheme that exploits cooperative downlink NOMA in D2D communications. One of the key advantages of BCD-NOMA over previous works is that it enables bidirectional D2D communications by utilizing downlink NOMA while also allowing two sources to utilize a single relaying node for data transmission to their destination nodes and thereby increasing its overall outage probability, ESC and EE performance. We derived the analytical expressions for the OP and ESC under both pSIC and ipSIC scenarios and verified them with the simulation results. Comprehensive results verified the effectiveness of the BCD-NOMA scheme in terms of OP, ESC and average EE through simulations and mathematical analysis over schemes such as OMA, hybrid CNOMA-OMA and other conventional schemes.

The performance of the BCD-NOMA system can be further enhanced through the dynamic power allocation scheme. In future, we plan to explore the game theoretical approach and artificial intelligence-based solutions to devise an efficient power allocation scheme for the proposed BCD-NOMA system.

## Figures and Tables

**Table 1:** Simulation Parameters.

Parameter	Symbol	Values
Distance between $S_{1}$ and *R*	$d_{S_{1}-R}$	0.25
Distance between $S_{2}$ and *R*	$d_{S_{2}-R}$	0.50
Distance between *R* and $D_{1}$	$d_{R-D_{1}}$	0.50
Distance between *R* and $D_{2}$	$d_{R-D_{2}}$	0.25
Distance between $S_{1}$ and $S_{2}$	$d_{S_{1}-S_{2}}$	0.20
Path Loss Exponent	*v*	4
Power Allocation Factor for NOMA	$a_{1}$	0.7
Power Allocation Factor for NOMA	$a_{2}$	0.3
Power Allocation Factor for NOMA	$a_{3}$	0.8
Power Allocation Factor for NOMA	$a_{4}$	0.2
Power Allocation Factor for NOMA	$b_{1}$	0.7
Power Allocation Factor for NOMA	$b_{2}$	0.3
Residual Interfering Signal	ξ	$10^{-4}$, $10^{-3}$, $10^{-2}$
$S_{1}$ Data Rate	$R_{S_{1}}$	0.50 bps/Hz
$S_{2}$ Data Rate	$R_{S_{2}}$	0.70 bps/Hz
$S_{1}$ D2D Data Rate	$R_{S_{1}-S_{2}}$	1.65 bps/Hz
$S_{2}$ D2D Data Rate	$R_{S_{2}-S_{1}}$	1.75 bps/Hz

## References

[1] Jiang W., Han B., Habibi M.A., Schotten H.D. (2021). The road towards 6G: A comprehensive survey. IEEE Open J. Commun. Soc..

[2] Ni W., Liu Y., Eldar Y.C., Yang Z., Tian H. (2022). STAR-RIS Integrated Nonorthogonal Multiple Access and Over-the-Air Federated Learning: Framework, Analysis, and Optimization. IEEE Internet Things J..

[3] Li K., Cui Y., Li W., Lv T., Yuan X., Li S., Ni W., Simsek M., Dressler F. (2022). When Internet of Things meets Metaverse: Convergence of Physical and Cyber Worlds. arXiv.

[4] Liu Y., Zhang S., Mu X., Ding Z., Schober R., Al-Dhahir N., Hossain E., Shen X. (2022). Evolution of NOMA toward next generation multiple access (NGMA) for 6G. IEEE J. Sel. Areas Commun..

[5] Xiao C., Zeng J., Ni W., Su X., Liu R.P., Lv T., Wang J. (2019). Downlink MIMO-NOMA for ultra-reliable low-latency communications. IEEE J. Sel. Areas Commun..

[6] Sun Y., Guo Y., Li S., Wu D., Wang B. (2018). Optimal resource allocation for NOMA-TDMA scheme with *α*-fairness in industrial internet of things. Sensors.

[7] Wei X., Al-Obiedollah H., Cumanan K., Zhang M., Tang J., Wang W., Dobre O.A. (2020). Resource allocation technique for hybrid TDMA-NOMA system with opportunistic time assignment. Proceedings of the 2020 IEEE International Conference on Communications Workshops (ICC Workshops).

[8] Choi J. (2016). On the power allocation for a practical multiuser superposition scheme in NOMA systems. IEEE Commun. Lett..

[9] Sarieddeen H., Abdallah A., Mansour M.M., Alouini M.S., Al-Naffouri T.Y. (2021). Terahertz-band MIMO-NOMA: Adaptive superposition coding and subspace detection. IEEE Open J. Commun. Soc..

[10] Rauniyar A., Engelstad P., Østerbø O.N. (2018). RF energy harvesting and information transmission based on NOMA for wireless powered IoT relay systems. Sensors.

[11] Liu Y., Yi W., Ding Z., Liu X., Dobre O.A., Al-Dhahir N. (2022). Developing NOMA to next generation multiple access (NGMA): Future vision and research opportunities. IEEE Wirel. Commun..

[12] Xia B., Wang J., Xiao K., Gao Y., Yao Y., Ma S. (2018). Outage performance analysis for the advanced SIC receiver in wireless NOMA systems. IEEE Trans. Veh. Technol..

[13] Zhang H., Fang F., Cheng J., Long K., Wang W., Leung V.C. (2018). Energy-efficient resource allocation in NOMA heterogeneous networks. IEEE Wirel. Commun..

[14] Rauniyar A., Østerbø O.N., Håkegård J.E., Engelstad P.E. (2022). Secrecy Performance Analysis of Cooperative Nonorthogonal Multiple Access in IoT Networks. IEEE Sensors J..

[15] Liu H., Ding Z., Kim K.J., Kwak K.S., Poor H.V. (2018). Decode-and-forward relaying for cooperative NOMA systems with direct links. IEEE Trans. Wirel. Commun..

[16] Zeng M., Hao W., Dobre O.A., Ding Z. (2020). Cooperative NOMA: State of the art, key techniques, and open challenges. IEEE Netw..

[17] Kim J.B., Lee I.H., Lee J. (2017). Capacity scaling for D2D aided cooperative relaying systems using NOMA. IEEE Wirel. Commun. Lett..

[18] Zou L., Chen J., Lv L., He B. (2020). Capacity enhancement of D2D aided coordinated direct and relay transmission using NOMA. IEEE Commun. Lett..

[19] Kai C., Wu Y., Peng M., Huang W. (2021). Joint uplink and downlink resource allocation for NOMA-enabled D2D communications. IEEE Wirel. Commun. Lett..

[20] Xu Y., Wang G., Li B., Jia S. (2019). Performance of D2D aided uplink coordinated direct and relay transmission using NOMA. IEEE Access.

[21] Budhiraja I., Kumar N., Tyagi S., Tanwar S., Guizani M. (2021). SWIPT-enabled D2D communication underlaying NOMA-based cellular networks in imperfect CSI. IEEE Trans. Veh. Technol..

[22] Cheng Y., Liang C., Chen Q., Yu F.R. (2021). Energy-efficient D2D-assisted computation offloading in NOMA-enabled cognitive networks. IEEE Trans. Veh. Technol..

[23] Budhiraja I., Tyagi S., Tanwar S., Kumar N., Rodrigues J.J. (2019). DIYA: Tactile internet driven delay assessment NOMA-based scheme for D2D communication. IEEE Trans. Ind. Informatics.

[24] Kader M.F., Islam S.R., Dobre O.A. (2021). Simultaneous cellular and D2D communications exploiting cooperative uplink NOMA. IEEE Commun. Lett..

[25] Kader M.F., Shahab M.B., Shin S.Y. (2017). Exploiting non-orthogonal multiple access in cooperative relay sharing. IEEE Commun. Lett..

[26] Alemaishat S., Saraereh O.A., Khan I., Choi B.J. (2019). An efficient resource allocation algorithm for D2D communications based on NOMA. IEEE Access.

[27] Yu S., Khan W.U., Zhang X., Liu J. (2021). Optimal power allocation for NOMA-enabled D2D communication with imperfect SIC decoding. Phys. Commun..

[28] Le M., Pham Q.V., Kim H.C., Hwang W.J. (2022). Enhanced resource allocation in D2D communications with NOMA and unlicensed spectrum. IEEE Syst. J..

[29] Zhao J., Liu Y., Chai K.K., Chen Y., Elkashlan M. (2017). Joint Subchannel and Power Allocation for NOMA Enhanced D2D Communications. IEEE Trans. Commun..

[30] Rauniyar A., Engelstad P.E., Østerbø O.N. (2020). On the performance of bidirectional NOMA-SWIPT enabled iot relay networks. IEEE Sensors J..

[31] Chrysologou A.P., Chatzidiamantis N.D., Karagiannidis G.K. (2022). Cooperative Uplink NOMA in D2D Communications. IEEE Commun. Lett..

[32] Zhang Y., Feng S., Tang W. (2021). Performance analysis of hybrid cellular and bidirectional device-to-device cooperative NOMA communication systems. IEEE Trans. Veh. Technol..

[33] Men J., Ge J., Zhang C. (2016). Performance analysis of nonorthogonal multiple access for relaying networks over Nakagami-*m* fading channels. IEEE Trans. Veh. Technol..

[34] Uddin M.B., Kader M.F., Shin S.Y. (2019). Uplink cooperative diversity using power-domain nonorthogonal multiple access. Trans. Emerg. Telecommun. Technol..

[35] Wei Z., Dai L., Ng D.W.K., Yuan J. (2017). Performance analysis of a hybrid downlink-uplink cooperative NOMA scheme. Proceedings of the 2017 IEEE 85th Vehicular Technology Conference (VTC Spring).

[36] Kader M.F., Shin S.Y. (2017). Coordinated direct and relay transmission using uplink NOMA. IEEE Wirel. Commun. Lett..

[37] Fang Z., Hu J., Lu Y., Ni W. (2019). Three-user cooperative NOMA transmission. IEEE Wirel. Commun. Lett..

[38] Yue X., Liu Y., Kang S., Nallanathan A., Ding Z. (2017). Exploiting full/half-duplex user relaying in NOMA systems. IEEE Trans. Commun..

